# Late Antique—Early Byzantine urban transformation in Mid-Northern Anatolia: A multiproxy approach from Pompeiopolis

**DOI:** 10.1371/journal.pone.0344987

**Published:** 2026-04-15

**Authors:** Melis Uzdurum, Mustafa Nuri Tatbul, Susan Marie Mentzer

**Affiliations:** 1 Department of Cultures, University of Helsinki, Helsinki, Finland; 2 Department of Archaeology, Ondokuz Mayıs University, Samsun, Türkiye; 3 History of Art, Bartın University, Bartın, Türkiye; 4 Senckenberg Centre for Human Evolution and Palaeoenvironment, Institute for Archaeological Sciences, Department of Geosciences, University of Tübingen, Tübingen, Germany; Universita degli Studi di Milano, ITALY

## Abstract

During the 6th century CE, many Late Antique cities in the eastern Mediterranean—especially in Anatolia—underwent major changes. By the 7th century CE, most had gradually lost their urban functions. As populations declined, urban spaces were reused for domestic, industrial and rural purposes. This shift is visible in the archaeological record, through the abandonment and reuse of public spaces. At Zımbıllı Tepe-Pompeiopolis, located in in Paphlagonia (northern Anatolia), which was occupied during Late Antiquity and Medieval periods, architectural remains, artifacts, and radiocarbon (C14) dates from both public and private areas show patterns consistent with this broader urban transformation across Anatolia between the 6th and late 7th centuries. To better understand how the site transformed over time, we conducted micro-scale, multiproxy analysis. These included macrobotanical and microdebris studies, micromorphology, x-ray fluorescence (XRF), microscopic fourier transform infrared (micro-FTIR), and C14 dating, based on samples from a street sewer and a connected private latrine. Together, these datasets reveal the cultural and natural processes that shaped the site’s last occupation, its abandonment, and its post-abandonment transformation. The disuse and infilling of the sewer suggest a halt in public maintenance toward the end of the occupation, while the latrine’s earlier abandonment reflects gradual organizational changes already in the 6th century. This study provides a macro-and-micro-scale view of urban transformation during the 6th–7th centuries using a multiproxy approach, which remains rare in archaeological studies of this period in Anatolia.

## Introduction

Historical, archaeological, and architectural data from the eastern Mediterranean—especially Anatolia—indicate a widespread pattern of urban decline and transformation in Late Antique cities beginning in the 6th century [1]. By the 7th century, many of these cities had largely lost their urban functions [2,3]. This transitional period and the following two centuries, often referred to as the “Dark Ages” due to limited archaeological visibility until the late 9th century [4], was followed by renewed prosperity during the Middle Byzantine period (9th–11th centuries). While palynological results support relatively favorable conditions and agricultural expansion during the Middle Byzantine period, the 6th–7th centuries experienced climatic instability associated with the Late Antique Little Ice Age [5–8].

**Fig 1:**
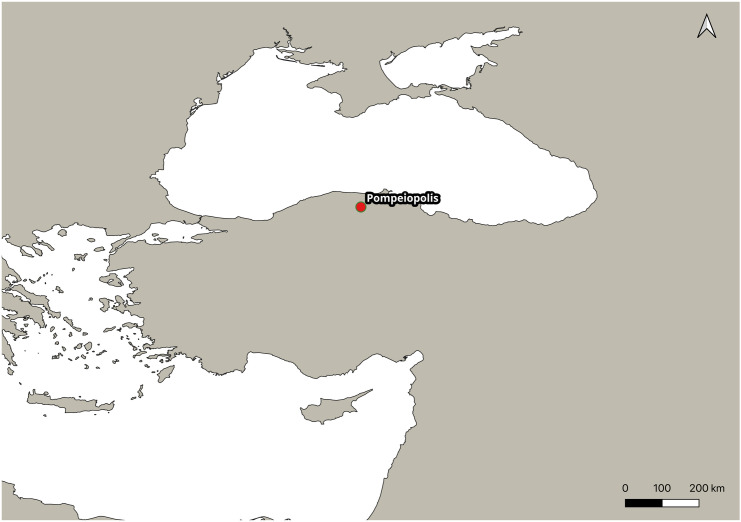
Location of Pompeiopolis in northern Anatolia. Basemap data from Natural Earth (public *domain, v. 5.1.1*). The map was prepared using QGIS (*v. 3.40.5-Bratislava*) (Credit: Mustafa N. Tatbul).

Urban decline has traditionally been linked to ruralization, either as its cause or consequence, shaped by intersecting social, political, economic, and environmental dynamics. Churches became the major public features in the urban infrastructure, and spread into the countryside, which is seen as an indicator of increasing ruralization [9]. Archaeologically, this transformation is visible in the abandonment and repurposing of public structures [3,10]. Facilities such as public baths and water management systems were gradually dismantled, and the production and circulation of characteristic material culture—such as coins and pottery—diminished [11]. Pompeiopolis, which is located in the northern Anatolia and occupied during Late Antiquity and Medieval periods (see Section 2 for phase overviews), imported wine amphorae (Fig 1). Trade in amphorae from the Levant ceased by the early decades of the 7th century, followed by Pontic amphorae by the mid-7th century [12]. The presence of Levantine wine amphorae in Pompeiopolis during the 5th-6th centuries, which indicates of an intensive and well organized long distance trade [13], ceased as a result of the fall of the maritime followed by the 7th century, a macro-phenomenon that was affected by macro-political changes in the Mediterranean. Looking at the fineware, the Pompeiopolis community turned to local products during the late 6th and early 7th century [14].

In general, these changes have commonly been attributed to the socio-political consequences of successive Byzantine–Persian and Byzantine–Arab wars, compounded by the loss of imperial territories and resources. Natural events—including earthquakes, plagues, famines, and climatic fluctuations—have also been considered contributing factors [15,16]. More recent, scholarship emphasizes adaptive responses and societal resilience, reframing this period not as one of collapse but of transformation [17].

Excavations at Zımbıllı Tepe-Pompeiopolis, have revealed a long occupation sequence extending from the mid-3rd to the 12th century, marked by significant transformations [18,19]. The settlement was extending on a conglomerate-sandstone-mudstone-limestone formation, having a rich alluvial zone proper for agricultural production.

**Fig 2:**
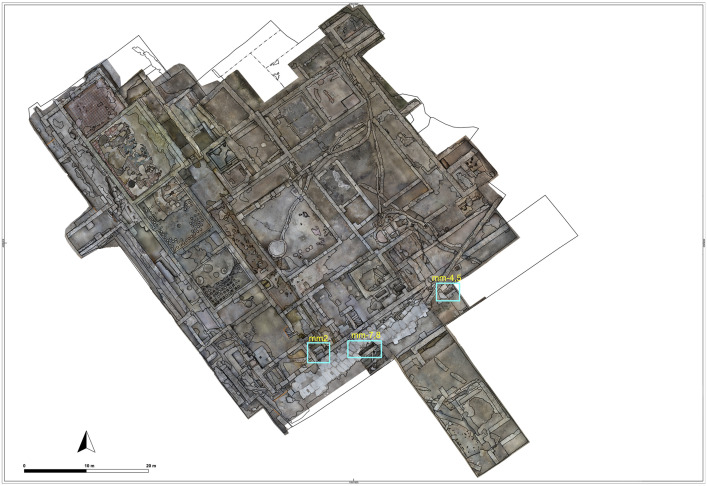
Architectural plan combined with orthophoto of the Domus showing the multiphased study area (Credit: Antonio Abate).

A large domus, constructed in the 3rd century CE on the city’s outskirts as a luxurious elite residence, measured at least 3,200 square meters, with roughly a third dedicated to reception areas (Fig 2). From the 6th century onward, the house was progressively subdivided and repurposed for the storage and processing of agricultural goods. In the 7th century, urban infrastructure such as water management and waste systems fell into disuse, and the settlement rapidly evolved into a rural landscape from the 8th century onwards, after a possible short hiatus, with a small agricultural hamlet persisting until its abandonment—likely following a violent event—in the 10th century. The subsequent construction of a Christian religious building with a small graveyard in the 11th century attests to a continued rural presence, while its intentional destruction in the 12th century may signal the region’s transition to Seljuk rule and its accompanying land reorganization.

This study focuses on the Domus as a private unit and the street sewer as a representative element of public infrastructure in order to assess whether Pompeiopolis conforms to the broader pattern of Late Antique urban transformation. In contrast to earlier narratives of abrupt collapse in the 7th century, recent approaches emphasize a more gradual process of survival and recovery between the 6th and 7th centuries [19,20]. Archaeological data from Pompeiopolis support this interpretation: the private latrine within the Domus ceased operation in the 6th century, followed by spatial reorganization within the house and a gradual decline of the sewer system in the second half of the 7th century. A small hamlet appears to have existed in the area between the 8th and 10th centuries. Rather than indicating complete abandonment, these changes suggest a shift toward a smaller-scale, domestically oriented, and rural occupation [21].

Comparable transformations have been noted in other western Anatolian cities. Uytterhoeven [22] argues that elite residences of the 4th–5th centuries were often subdivided into smaller units with industrial or rural functions around the mid-6th century, inhabited by lower social strata [23]. Architectural analysis of the Pompeiopolis Domus confirms this trend: the house lost its elite features from the 6th century onwards, yet urban infrastructure—such as the sewer—remained functional into the later 7th century.

The collection of paleoenvironmental proxies from the 2009–2023 excavation campaigns offer a unique body of evidence, not only due to its near-millennial span but also through its close integration with socially and architecturally contextualized data [21,24]. This study examines 6th–7th century contexts at Pompeiopolis, focusing on the decline of the Domus and the cessation of urban infrastructure recorded in water-related features such as supply ducts, house latrines, disposal drains, and the main sewer. Using a multiproxy, macro- and micro-scale approach—combining macrobotany, micromorphology, micro x-ray fluorescence (micro-XRF), microscopic fourier transform infrared (micro-FTIR), polarized energy dispersive x-ray fluorescence (PED-XRF), and radiocarbon dating—we investigate the cultural and natural formation processes evident in the stratigraphic sequences. Key questions include the final use, maintenance cessation, and mechanisms of infill in these features: can the stratigraphic records of the latrine and sewer be correlated, and do they reflect direct interaction? Rather than interpreting these changes as abrupt abandonment, we assess evidence for gradual shifts in maintenance, use, and environmental context, offering new insights into ruralization and urban resilience in Late Antique Anatolia.

### Archaeological context and overview

#### The late Antique Domus.

Around the middle of the 3rd century, a grand house was built on the edge of the ancient city, in an area developed through a system of perpendicular road axes [18,21,24]. The Domus occupies two blocks of the urban grid and is articulated across at least two levels along the hillside. A northern reception court provides access to the residential part of the house, organized around a central peristyle, and—via a monumental staircase—to a suite of representative rooms located at a higher level. The southern sector of the house, aligned with the main paved road climbing the hill, is occupied by service premises, where both the latrine and kitchen have been identified, while a large entrance allows access from the road for carts. The southern side of the peristyle hosts a fountain-nymphaeum likely topped by a cistern fed by pressurized terracotta pipes. The scale of the building and the richness of its decorations, mostly mosaic floors and marble revetments, reflect the elite status of its owners. Over more than two centuries, many renovations occurred, including new mosaic floors and a complete reconstruction of the northwestern corner of the peristyle portico. All these interventions served to embellish and enhance the functionality of a prestigious residence, within what was still an active urban context—as evidenced by the paving of the southern main road and the construction of an urban sewer collector.

#### The transformation of the Domus.

In the second half of the 6th century, a progressive transformation of the Domus was recorded [18,21,24]. The interior spaces were divided into smaller rooms, with evidence of craft activities and the establishment of storage facilities. The hypocaust heating systems were deactivated, and the central garden was built over with new structures. The fountain-nymphaeum continued to function, albeit now supplied by a new aqueduct, and the wastewater disposal system was completely reorganized. Rainwater collected in the western garden was directed into a cistern adapted from a former hypocaust. During this phase, the latrine was closed to make room for baking installations, likely replaced by a simpler facility linked to the fountain-nymphaeum’s drainage. These changes continued—though unevenly—across the entire house until the late 7th century, when the collapse of original roofs and more drastic alterations to the wall structures marked a full redefinition of the building’s identity. This coincides with the disuse of the urban sewer and the gradual covering of the paved road with dirt and rubble—clear signs of the absence of municipal oversight, at least in this part of the town.

#### The early Byzantine hamlet.

As a result of these changes, the townscape defined during the 2nd and 3rd centuries was replaced by a rural landscape by the early 9th century, characterized by different forms of housing and land use until its abandonment in the 12th century [18,21,24]. Although the archaeological evidence is limited, excavations suggest a small settlement of isolated buildings—likely multistory—arranged around a central open area containing large storage jars. Characterization of the phase with numerous pithoi (possibly of private use) [25] could be taken as an indicator that the small community relied heavily on agricultural production and self-sufficiency. These new buildings spread indiscriminately across previously public and private spaces, reinforcing the idea of an administrative break. Nevertheless, the substantial leveling work on the earlier ruins’ points to the continued presence of a managing authority in the area.

#### The middle Byzantine chapel.

In the 10th–12th centuries, the hamlet was abandoned—possibly after a destructive event. Soon afterward, a quadrangular building was constructed in the area [18,21,24]. Lime for the construction was produced onsite in a lime kiln using materials from the surrounding ruins.

Along the southern side of the building, about ten burials were installed using stone and brick, oriented east–west. Some were multiple burials, and two contained grave goods that allowed dating to the 11th–12th centuries. Among these finds was an inscribed cross-shaped encolpion, which—together with other artifacts—suggests that this building functioned as a Christian countryside chapel [26–29]. Its religious function may also have led to its intentional destruction in the 12th century.

#### The late rural use.

After the destruction of the chapel, likely corresponding to the Seljuk conquest of the region, subsequent activity was limited to terracing and/or boundary-marking interventions for agricultural use [18,21,24]. Occasional spoliation is recorded, mainly for the recovery of fired bricks from earlier structures.

#### Absolute chronology.

As mentioned above, this paper focuses on contexts dated by radiocarbon (C14) analysis to the transformational period of the 6th and 7th centuries. These include the fillings of the latrine in the southern sector of the Domus and the main street sewer. Although both contexts reflect the neglect of hydraulic infrastructure, they differ in chronology and formation processes, as clarified by the analyses presented in this paper. Radiocarbon samples were analyzed by the Tübitak Marmara Research Center. An accelerator mass spectrometer produced by National Electrostatics Corporation (Model 3SDH-1) was used for the analyses. Radiocarbon dates were calibrated with OxCal v4.4.4 [30,31].

Four C14 charcoal samples were taken from selected stratigraphic units: Sample 5 from US 1808 (latrine), Samples 6 and 7 from two different locations in US 1414 (sewer), and Sample 8 from US 1833 (sewer).

**Fig 3:**
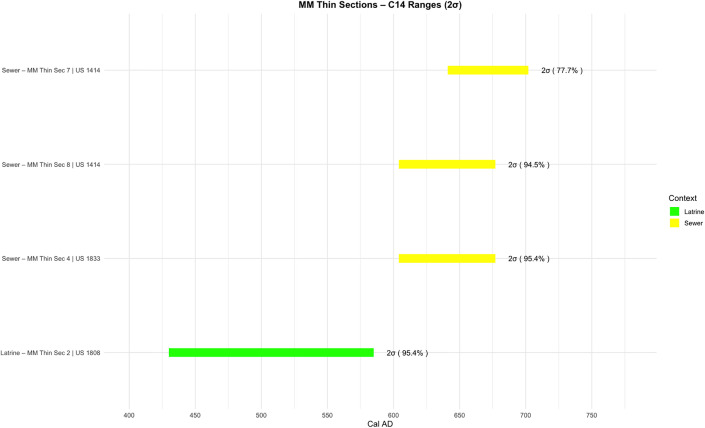
Graph representing C14 sample dates from sewer and latrine contexts (Credit: Mustafa N. Tatbul).

Sample 5 (latrine) is dated to 430–585 CE (95.4%); Sample 6 (sewer) to 582–657 CE (95.4%); Sample 7 (sewer) to 641–702 CE (77.7%); and Sample 8 (sewer) to 604–677 CE (94.5%) (Fig 3, S1 Table). Based on the highest probability ranges, the final use of the latrine falls between the 5th and 6th centuries, whereas all sewer samples cluster in the 7th century. These results confirm that the sewer continued in use after the latrine was abandoned, consistent with architectural phase changes identified during the excavation.

A single identifiable coin (Justinian I, 527–565 CE; follis 540/541 CE) recovered from the latrine further supports mid to the late 6th century date for its final use.

## Materials and methods

**Fig 4:**
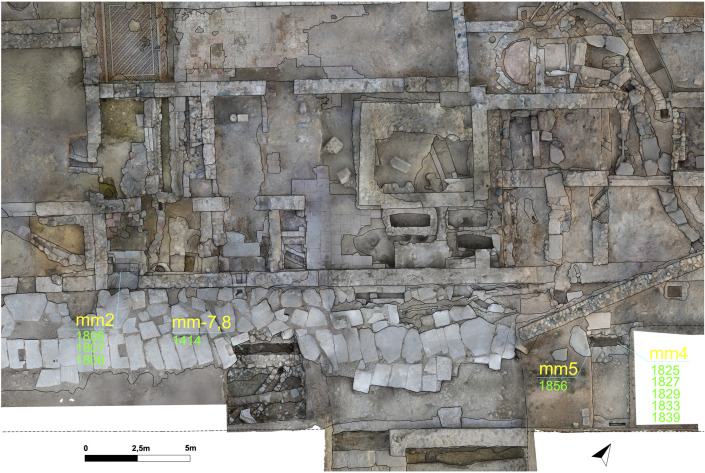
Architectural plan combined with orthophoto showing the stratigraphical units and sampling locations (Credit: Antonio Abate).

In addition to the architectural assessment of the archaeological context, this study adopts a macro – micro-scale, multiproxy approach involving four main methods. This section outlines the sampling strategy, analytical procedures, and interpretive potential of each method (Table 1). Also see (Fig 4) for spatial information of the stratigraphical units and sampling locations.

**Table 1:** Sample identifications and their contextual relationship.

Stratigraphical units defined by the excavators (top-down)	Flotation Sample ID	Micromorphology Thin Section ID	Microunits^a^ interpreted based on the Micromorphology Thin Sections (top-down)	PED-XRF ID	C14 ID	Context
1806	54	2	1	1		Latrine
1807	55	2	2	2, 3		Latrine
1808	56	2	3	4	5	Latrine
1825	59	4	1	5		Street sewer
1827	60	4	2	6		Street sewer
1829	61	4	3	7		Street sewer
1833	62	4	4	8	8	Street sewer
1839	63	4	5	9		Street sewer
1856−1	–	5	1, 2	10		Street sewer
1856−2	–	5	3, 4	11		Street sewer
1856−3	–	5	5, 6	12		Street sewer
1414	21	7	1	14	6	Street sewer
1414	21	7	2, 3	15	6	Street sewer
1414	19	7	4, 5, 6	16	7	Street sewer
1414	19	7	7, 8	17	7	Street sewer
1414	21	8	1	19	6	Street sewer
1414	19	8	2	20	7	Street sewer

^a^We use “microunit” to describe sedimentary subunits visible in thin section.

No human remains were analyzed in this study. All archaeological materials (soil and sediments, and associated micro-remains) derive from controlled excavations at Pompeiopolis. The material is curated under the authority of the Pompeiopolis Excavation Directorate and the Kastamonu Museum Directorate (Kastamonu, Türkiye). All necessary permits were obtained for the described study, which complied with all relevant national regulations. Fieldwork, sampling, and analysis permissions were granted by the Ministry of Culture and Tourism of the Republic of Türkiye to the Pompeiopolis Excavation Directorate.

### Macrobotanical (light fraction) and microdebris (heavy residue) analyses

A total of 11 flotation samples were collected from the fills of the sewer and latrine, corresponding to 5 micromorphology blocks, and 17 loose sediment samples from the same contexts (Table 1). Both light fractions (LF) and heavy residues (HR) were sorted, identified, and quantified. In total, 207 liters of soil were processed. The sampling strategy aimed to ensure parallel and corresponding samples across all methods. No strict soil volume was applied; rather, it depended on the condition and availability of the archaeological fills.

Flotation was performed using a Siraf-type barrel system with dual intake—from a water source and an air compressor. Air bubbles released through the holes of an S-shaped pipe beneath a 1 mm plastic mesh loosened the soil and allowed the LF to rise. A 100-micron tulle cloth (netting) was stretched over a perforated bucket to collect the floating LF as water drained away. The sunken HR was collected separately for analysis. Soil volume was measured prior to flotation using a scaled plastic bucket, allowing material density per liter to be calculated. Specimen counts were normalized per liter to evaluate density and spatial patterns across sampling contexts.

Once dried, LF samples were sieved using 2 mm, 1 mm, and 0.5 mm mesh sizes. Plant remains were sorted and identified under a Celestron Labs Stereo Microscope (10 × –60 × magnification) and documented using a Tomlov TriL110 digital camera, with reference to standard seed atlases [32–35]. Insect remains were counted—although not identified to species—in order to assess their density across stratigraphic units. These remains were preserved for future specialist analysis.

All HR materials between 3 cm and 1 mm were sorted and identified [36]; fractions smaller than 0.5 mm were kept for future study. Recovered material included glass, metal, tesserae, glass and metal slag, ceramics, and bone fragments of medium- to large-sized animals [37]. Bird, rodent, and fish bones were separated and preserved for later specialist identification.

### Micromorphology (thin section analysis)

Micromorphological analysis focused on identifying both anthropogenic and natural components within the sewer and latrine fills, particularly features such as human waste and post-abandonment sediment accumulation [38]. Sampling was conducted from well-preserved areas of the deposits, avoiding layers with excessive stones. Blocks were cut with a knife and stabilized using plaster bandages before removal.

Thin section preparation was performed at Hacı Bayram Veli University (Ankara). Samples were cut with a rock saw, dried at 60 °C, and impregnated for one hour with Araldite AY 103−1 and Hardener HY 991 epoxy. After hardening, blocks were trimmed to 9 × 12 cm, re-coated, polished with 600-grit powder, and mounted on custom-cut glass slides. Sections were then ground to 30 microns and left uncovered for analysis.

High-resolution scanning was conducted at the University of Tübingen using an Olympus petrographic microscope with Prior motorized stage, connected to a desktop computer. Overlapping photomicrographs were captured under plane- (PPL) and cross-polarized light (XPL) at 5 × magnification using a Basler camera, and stitched into.SVS-format mosaics, using the Microvisioneer Auto-WSI software. SedeenViewer – a software program for viewing SVS files – allowed remote, high-resolution observation of the samples.

Microscope work at the Microscopy & Geoarchaeology Laboratory (Tübingen) employed a Zeiss Stemi 2000-C stereomicroscope and a Zeiss AxioImager A2 petrographic microscope, both with digital imaging. Observations were carried out under PPL and XPL using Zeiss Axiovision software. Sample descriptions addressed porosity and voids, sediment texture and sorting, coarse/fine (c/f) fraction relationships, fabric, pedofeatures, and microstructure [39,40]. The coarse/fine (c/f) limit was set at 10 µm [39]. Additional observations included aggregates, inclusions (biogenic and anthropogenic), and diagnostic features related to source and depositional processes. Micromorphological descriptions follow the internationally accepted terminology of Stoops [39].

### Micro-XRF and micro-FTIR

Micro x-ray fluorescence (micro-XRF) was employed to visualize and describe the spatial distribution of major and trace elements within the thin sections [41,42]. In total, thirteen elements were analyzed (Al, Si, K, Ca, P, Fe, Mg, Na, S, Cl, Ti, Mn, Cr). This approach was not intended for basic identification—which optical microscopy already provides—but rather to complement micromorphological observations by chemically confirming materials identified in thin section (e.g., fecal remains, lime-related features and Fe and Mn redox pedofeatures). These same elements (Ca, P, Fe, Mn for anthropogenic inputs and Al, Si, K, Fe, Ca for geogenic components) also form the basis of the PED-XRF interpretation presented in the following section.

Analyses were conducted on uncovered thin sections at the Microanalytics Laboratory at the University of Tübingen using a Bruker M4 Tornado micro-XRF analyzer. Mapping was performed under full vacuum with a 20-micron spot size, pixel spacing of 30–90 microns, and a dwell time of 10 ms. The interaction with the x-ray beam, generated by a rhodium tube, was captured with a dual-detector system and processed using Bruker Esprit software.

Microscopic fourier transform infrared (micro-FTIR) spectroscopic analyses were conducted following both optical petrography and micro-XRF for the purpose of mineralogical identifications. Analyses were conducted in reflectance mode directly off the surface of the thin sections using an Agilent Cary 610 microscope attached to a Cary 660 bench. The resulting spectra – collected at 2 cm-1 resolution with 64 co-added scans – were compared to an in-house reference database of archaeological materials and minerals in thin section.

### PED-XRF analysis

XRF analysis was conducted on loose sediment to obtain quantitative chemical data for statistical evaluation, including hierarchical cluster analysis (HCA) and principal component analysis (PCA), enabling comparisons between different depositional contexts [43,44]. All statistical analyses—including PCA, HCA, and descriptive tools such as boxplots—were performed using RStudio (version 4.5.0).

After pulverization in an agate mortar, the loose samples were pressed into 32 mm disks, mixed with wax, and analyzed using a a polarized energy dispersive x-ray fluorescence (PED-XRF) spectrometer (X-lab 2000). This instrument can detect elements ranging from sodium (Na, atomic number 11) to uranium (U, atomic number 92), with detection limits of approximately 0.5 ppm for heavy elements and 10 ppm for lighter elements. In total, 48 elements were identified, with major and minor elements reported as oxide percentages (%) and trace elements expressed in parts per million (ppm).

An initial PCA included 14 chemical oxides. Prior to multivariate analysis, an exploratory variance assessment was conducted. Elements with very low variance (var < 0.01) were excluded due to limited discriminatory power and proximity to detection limits (Na₂O, SO₃, Cl, V₂O₅, Cr₂O₃, MnO, TiO₂). P₂O₅ and K₂O, despite medium variance, were retained for their archaeological relevance. A revised PCA was then performed using the reduced dataset. The retained elements—CaO, P₂O₅, SiO₂, Al₂O₃, Fe₂O₃, and K₂O supporting their relevance and cross-methodological consistency.

## Results and discussion

### Macrobotanical (LF) and microdebris (HR) data

Observing the recovery conditions, all cultivated plant species were found in charred forms, with the exception of *Galium sp.* as a crop weed. Different levels of the latrine context contained more cultivated taxa than the street sewer levels (S2 Table). This ratio was also observed in prior comparison of microdebris samples taken from the street sewer and internal channel contexts of the Domus, and the low number of charred seeds was explained as being due to the weathering conditions. Specifically, the stronger flow rate of the street sewer may have contributed to fragmentation, while internal channels could have had a less destructive impact on the charred plant materials. Charred plant seeds could also be more abundant and intact in the internal channels due to being closer to the original location of burning and discard activities. Even though the number of botanical samples was very small, the sewer had only wheat and grape, while the latrine had all three (wheat, barley, grape) plant groups. The upper levels of the latrine had more variety compared to the lower parts of the latrine and the sewer context. *Galium sp.*, as a crop weed, was observed in both contexts. Presence of charred seeds and charcoal in latrine and further in the sewer contexts could be related to dumping of cooking waste to latrine for odor reduction and hygiene [45]. Presence of animal bone fragments in the heavy residue of the latrine may support this interpretation (S3 Table).

**Fig 5:**
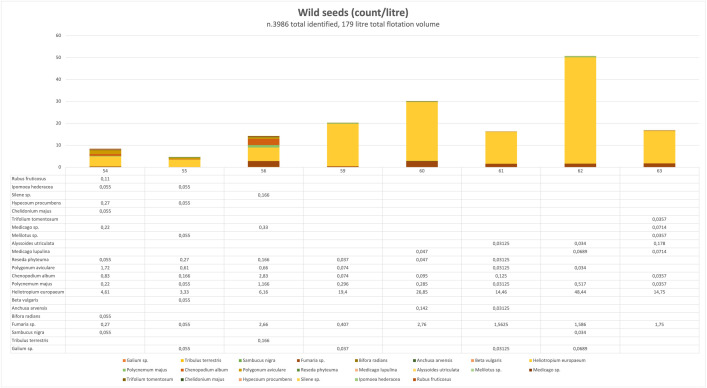
Distribution of wild taxa among sampling contexts (Credit: Mustafa N. Tatbul).

All wild taxa were found either in mineralized or water-logged conditions (Fig 5). The deposits from the latrine and sewer contexts appear to be predominantly phosphatized, characterized by calcium-phosphate mineral replacement, as suggested by their distinct color and microtextural properties. Under certain conditions—particularly in environments enriched with lime or other calcium-bearing materials—calcification may also occur, contributing to additional pathways of calcium-phosphate substitution within the structural tissues of plant remains [46–50]. Lithological structure of Zımbıllı Tepe is also rich in limestone, thus has potential to cause calcification of the plant materials.

Twenty-three different species of wild taxa were identified. The weed assemblage reflects both ruderal and segetal environments, pointing to inputs from waste-rich areas and cultivated fields [51–53]. Ruderal species such as *Chenopodium album*, *Polygonum aviculare*, *Heliotropium europaeum*, and *Sambucus nigra* thrive in dung-enriched soils, trampled ground, and refuse deposits, conditions consistent with a latrine or other heavily disturbed setting. At the same time, segetal weeds including *Fumaria sp.*, *Lithospermum arvense*, and *Polycnemum majus* are characteristic of arable land and cereal fields, indicating that crop-processing debris or soil from manured fields also contributed to the deposit. Together, these taxa suggest a mixed, strongly anthropogenic origin for the material.

**Fig 6:**
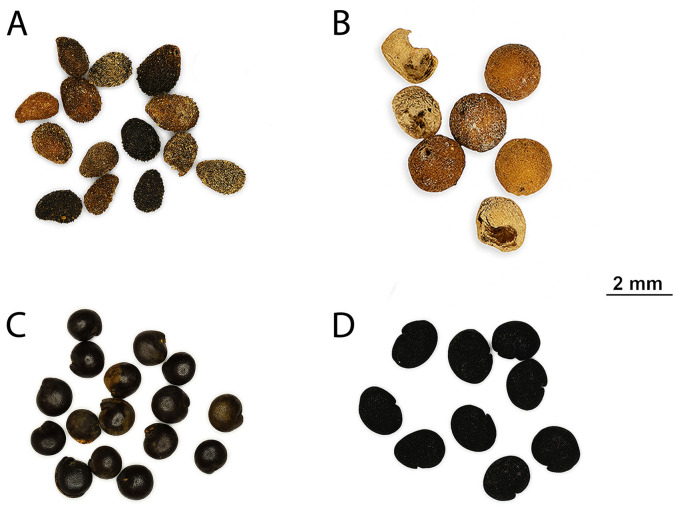
Wild seed remains recovered from latrine and sewer contexts: (A) *Heliotropium* sp.; (B) *Fumaria* sp.; (C) *Chenopodium album*; (D) *Polycnemum majus* (Credit: Mustafa N. Tatbul).

The latrine context had a greater variety of plant remains than the street sewer. Six taxa were present in all stratigraphic levels and contexts, but obviously dominant in all sewer levels. Among them are *Heliotropium sp.*, *Fumaria sp.*, *Polycnemum majus*, *Chenopodium album*, *Polygonum sp.*, and *Reseda phyteuma* (Fig 6). This pattern indicates that these species were consistently present in the past environment and entered the archaeological record throughout the occupation, abandonment and post-abandonment phases. *Heliotropium sp.* and *Fumaria sp.* are frequently present in archaeological records and are often interpreted as possible medicinal herbs. Their abundance could also be related to their properties as being rich seed-releasing taxa. The rational minority of cultivated charred taxa vs. the wild taxa could be explained by the immense seed release of nature of the latter while the source of cultivated taxa is dependent on fire-related human activity mostly. The latrine and sewer contexts are irrelevant for considering the charred seeds as in situ, but rather in secondary contexts, finding their way as discard behavior (i.e., sanitation) [54].

**Fig 7:**
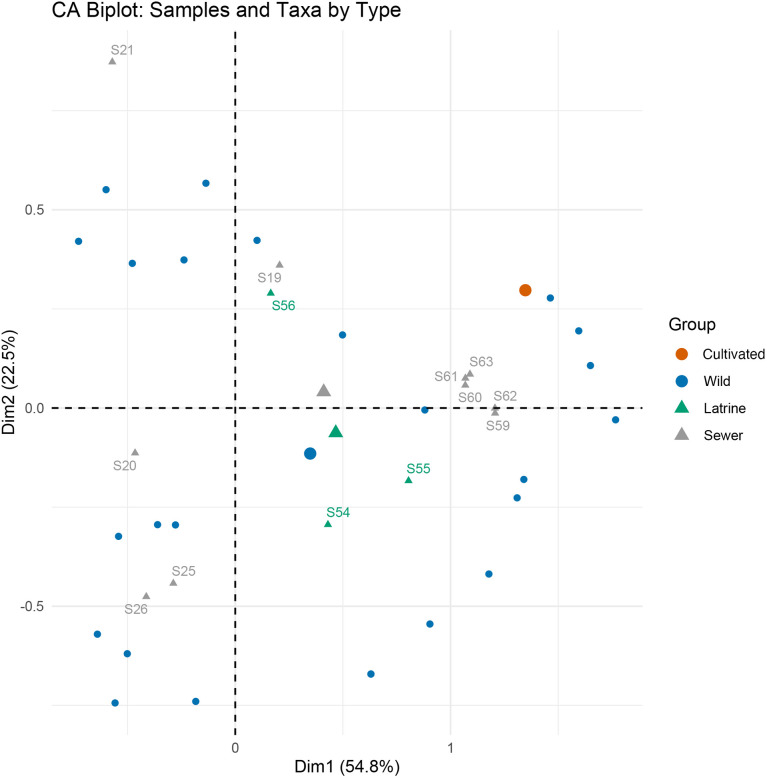
Correspondence analysis (CA) biplot of macrobotanical data showing the separation of latrine and sewer contexts. The distribution of plant taxa by type (cultivated vs. wild) highlights context-based depositional differences (Credit: Mustafa N. Tatbul).

Correspondence analysis of the macrobotanical sample distribution (Fig 7) shows a clear separation between latrine and sewer contexts along Dimension 1, which explains the majority of variance (54.8%). Latrine samples cluster separately from sewer samples, and this pattern corresponds well with the dominance of charred cultivated taxa observed in the latrine fills. The biplot also shows the positioning of plant taxa by type (cultivated vs. wild), further supporting the interpretation of context-based depositional differences.

The latrine samples from top to bottom (s 54–56) had 17 different types of wild seeds. *Heliotropium sp.* was the dominant species in all three levels of the latrine (Fig 6A). *Polygonum sp.* was abundant in the upper part, and present in similar amounts in the middle and bottom levels of the fill. *Tribulus terrestris*, *Fumaria sp.* and *Polycnemum majus* were mostly concentrated at the bottom of the latrine fill. *Heliotropium sp.* was the dominant species at all levels of the street sewer from top to bottom (s 59–63) corresponding to micromorphology sample 4, with an abundance at the bottom almost double that of the rest of the sequence. *Fumaria sp.* is the second most abundant type at all levels (Fig 6B).

*Fumaria sp. Heliotropium sp.* and *Chenopodium album* were the common abundant species at all levels of the sewer corresponding to micromorphology samples 7 and 8 (Fig 6C). *Polycnemum majus*, *Polygonum sp.*, *Lithospermum arvense* and *Anchusa arvensis* were mostly abundant in the middle level of the sewer (*Chenopodium album* -fat hen- is a consumable potherb weed, however self-grown in watercourses, see [55,56], for the high nutritional and caloric value of seeds) (Fig 6D).

Within the HR, artifactual and ecofactual materials smaller than three cm were sorted in the flotation samples. Ceramics and bone fragments were the most abundant in all sampling contexts (S3 Table), as expected in most archaeological deposits. Other fragments were of metal and glass objects. Metal fragments were mostly in lower sewer contexts. Sewer samples 59–63, corresponding to micromorphology sample 4, have the greatest density of ceramic fragments. Bone fragments were most abundant at the lower levels of the sewer (s 19, 60–62). Latrine samples (s 54–56) have the lowest bone density. Glass density shows a similar pattern with the ceramics being high in samples 19, 60, 62 and middle latrine level (s 55).

### Micromorphology

Most samples display sandy matrices that are generally well sorted and primarily composed of quartz, feldspar, carbonates, and opaque minerals derived from the Zımbıllı bedrock [18], although some silty and silty-clay microunits with variable sorting are also present (Table 2). Local rock clasts such as sandstone, fossiliferous limestone, and quartzite occur throughout, alongside common anthropogenic materials including ceramic and/or terracotta fragments, charcoal, bone, and construction debris.

**Table 2:** Micromorphological characteristics of thin sections analyzed in the study.

Sample ID	Micro unit	Thickness (cm)	Texture	Sorting	c/f related distribution	Voids	Microstructure
2	1	4	coarse sand-sized	poor	porphyric	channel and planar	angular blocky
			silt	well			
	2	7	fine sediment with clay			channel and chamber	
			sand	poor			
			silt	well			
	3	microunit not represented in the thin section due to sample disruption during laboratory preparation.					
4	1	4	sand-sized	well	porphyric	channel and chamber	channel
	2	1	sand-sized	poor		channel	channel
	3	5	silty-clay	well		vugh	spongy
	4	1	sand-sized	poor		channel	channel
	5	2	sand-sized	well		vugh	spongy
5	1	3	fine	well	porphyric	channel, planar, vesicle	massive
	2	3	coarse	poor			
	3	1	fine	well			
	4	1,5	coarse	well			
	5	1	fine	well			
	6	1,5	coarse	well			
7	1	4	coarse	poor	porphyric	channel, vesicle	channel
	2	0.5	coarse	poor			
	3	1	fine	poor			
	4	1	coarse	well			
	5	0.5	coarse	poor			
	6	1	fine	well			
	7	1	coarse	well			
	8	1.5	fine	poor			
8	1	5	coarse	poor	porphyric	channel, vesicle	massive
	2	6	fine	poor			

#### Latrine.

**Fig 8:**
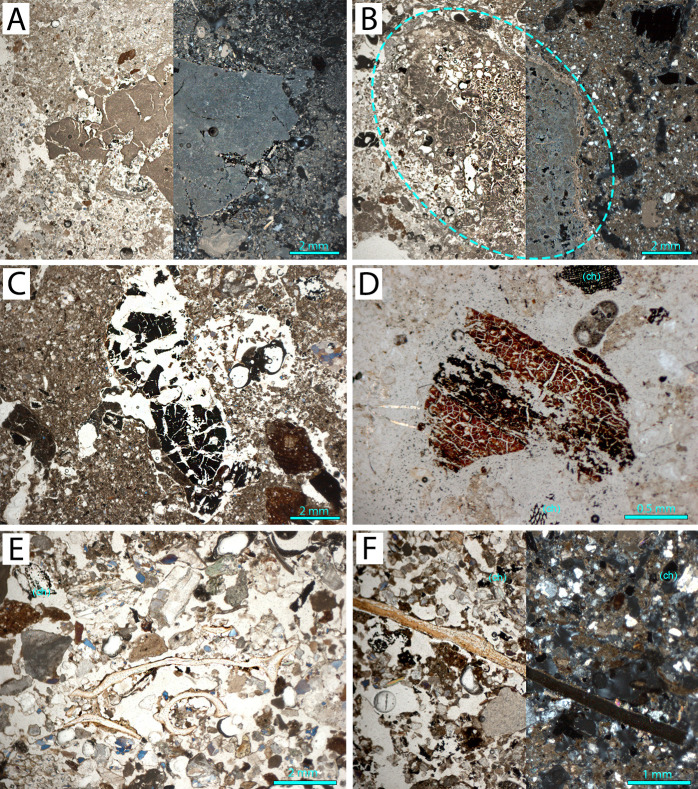
Micromorphological evidence for lime, charcoal, and biogenic inclusions across contexts: (A) Large fragment of fired lime with internal planar voids (S.2, PPL left, XPL right), suggesting possible over burning. (B) Reacted lime (S.7, PPL left, XPL right). (C) Charred cereal grain embedded in the matrix (S.8, PPL). (D) Organic material (possibly leather) with shrinkage planar voids; charcoal (ch) marked with (S.4, PPL). (E) Fish bone with phosphatized areas and charcoal (ch) fragment (S.7, PPL). (F) Coprolitic bone fragment with phosphate alteration causing complete isotropy under XPL and charcoal (ch) pieces (S.7, PPL left, XPL right) (Credit: Melis Uzdurum).

The latrine fill contains abundant sand-sized ceramic and terracotta fragments together with dominant fired lime. Under high magnification, the fired lime fragments—identified by their fine texture relative to cement and bluish interference colors in XPL (Fig 8A)—are embedded within fecal and organic material. Planar voids (aka shrinkage cracks or shrinkage fractures [57]) visible in these fragments indicate that some lime was overcooked, making it less suitable for plaster or mortar production. These fragments likely represent quicklime (CaO) applied to reduce odor and maintain sanitation. Occasional charred cereal grains (likely barley or wheat) and wood charcoal are present (Fig 8C). Rare possible human fecal remains embedded in lime-rich aggregates suggest preserved waste material from the period of active use. These coprolitic aggregates show an amorphous, yellowish-brown, optically isotropic Ca–P groundmass with diffuse, unstructured internal fabric and associated degraded organic matter. Their occurrence within a purpose-built latrine strongly supports a human origin. Microstructures reveal internal crusts, calcitic coating, and shrinkage planar voids (shrinkage cracks) in organic material—evidence of episodic moisture and drying cycles.

#### Sewer.

In the sewer samples, ceramics occur in a broader size range—from gravel to fine sand—and are particularly dense in Samples 5 and 8, where they show angular forms and occasional weathering. Reacted lime and phosphate-rich lime are especially common in Sample 7 (Fig 8B), suggesting exposure to moisture and possible biological degradation. This pattern points to slaked lime (Ca(OH)₂) or lime transformed through prolonged moisture exposure, perhaps via redeposition or runoff.

Charcoal content varies widely. Samples 7 and 8 include thick charcoal-rich layers (e.g., microunits 2 and 5 in S.7) that are often associated with fish bones, shell fragments, and phosphatized bone (Fig 8E and 8F). Sample 8, though sharing horizon and depositional phase with Sample 7, contains less charcoal but includes coarse gravel-sized pieces showing phosphate decomposition, possibly due to sustained wet conditions in the sewer. Biogenic inclusions such as insect excrement and possible leather fragments were observed in Samples 4 and 5 (Fig 8D). Sample 7 also contains phosphatized bone interpreted as coprolitic in origin (Fig 8F), supporting waste-related material transport.

**Fig 9:**
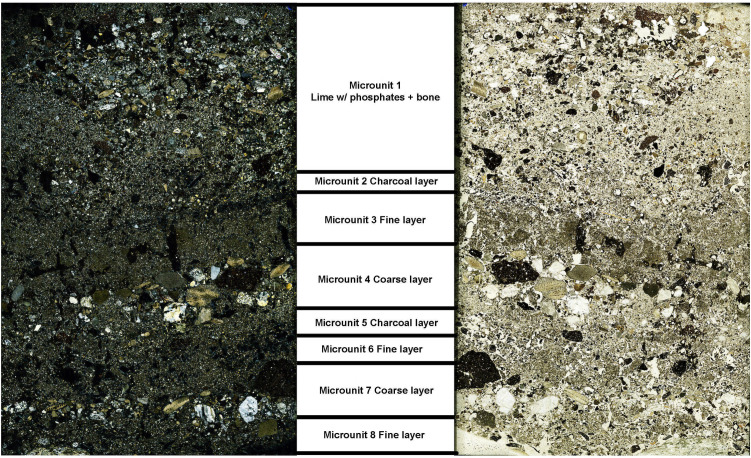
Repetitive coarse–fine–charcoal layering observed in thin section Sample 7, indicating rhythmic sedimentation likely associated with fluctuating water flow in the sewer context (PPL left, XPL right). Note that the two images are mirrored (Credit: Susan M. Mentzer).

Sewer microstructures are more complex than those of the latrine: laminated and massive microstructures appear in Samples 4 and 5; charcoal-rich microunits of Sample 7 exhibit channel microstructures; and Sample 8 shows massive structures with porphyric c/f distribution. Sample 5 contains both primary and secondary iron oxide accumulations—often surrounding limestone or clay aggregates—likely formed at the interface between anoxic sediment and oxygenated water, reflecting periodic wetting and drying episodes, possibly linked to fluctuating water levels within the sewer. A particularly diagnostic feature is the repetitive coarse–fine–charcoal layering in Sample 7, indicating rhythmic sedimentation driven by fluctuating low- and high-energy water flow (Fig 9). This pattern is consistent with the sewer’s drainage function.

### Micro-XRF and micro-FTIR

Micro-XRF elemental mapping was applied in the latrine and sewer contexts to illustrate elemental distributions and refine depositional interpretations, of the 13 elements analyzed, 6 were selected for illustration here with the distribution of geogenic matrix elements (Al, Si and K) plotted in grey and the more variable Ca, P and Fe represented in colors.

**Fig 10:**
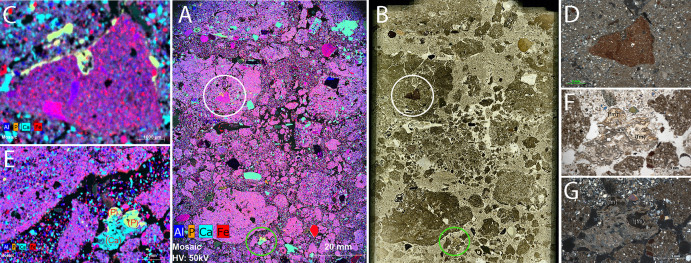
Micromorphological and micro-XRF evidence for phosphate–lime interaction and fecal waste indicators in Sample 2. The center of the image plate shows the PPL scan of the thin section next to an overview elemental map while the perimeter images show details of circled areas. (A) Elemental map (micro-XRF) of Al, P, Ca and Fe. (B) High-resolution scan of the same thin section. White circles mark ceramic or terracotta fragments; green circles indicate areas of degraded possibly human excrement mixed with reacted lime. (C) Detail elemental map from the white-circled area showing Ca and P enrichment around a ceramic fragment which appears here as yellow. (D) Micromorphological image of the same ceramic fragment surrounded by Ca–P precipitates (identified using micro-FTIR as hydroxylapatite). (E) Elemental map from the green-circled area highlighting a material that grades from aqua to yellow, indicating that one area contains only Ca within the mineral calcite, while the other contains both Ca- and P within hydroxylapatite, which is attributed to an amalgamation of excrement and reacted lime. (F and G) Thin section images of the same material under PPL (F) and XPL (G), showing an optically isotropic P-rich phase, consistent with degraded fecal material (marked as [fm]) (Credit: Melis Uzdurum).

Elemental mapping of Sample 2 (latrine) reveals a clear co-localization of Ca and P around ceramic fragments (Fig 10A and 10D) and within lime-rich aggregates (Fig 10E and 10G). These occur as irregular patches with discontinuous laminae, indicating episodic waste disposal followed by rapid infilling. Elevated Ca points to direct lime application for sanitation, while P reflects the mineralization of organic waste. Fe appears only as fine, scattered halos and remains secondary in abundance.

**Fig 11:**
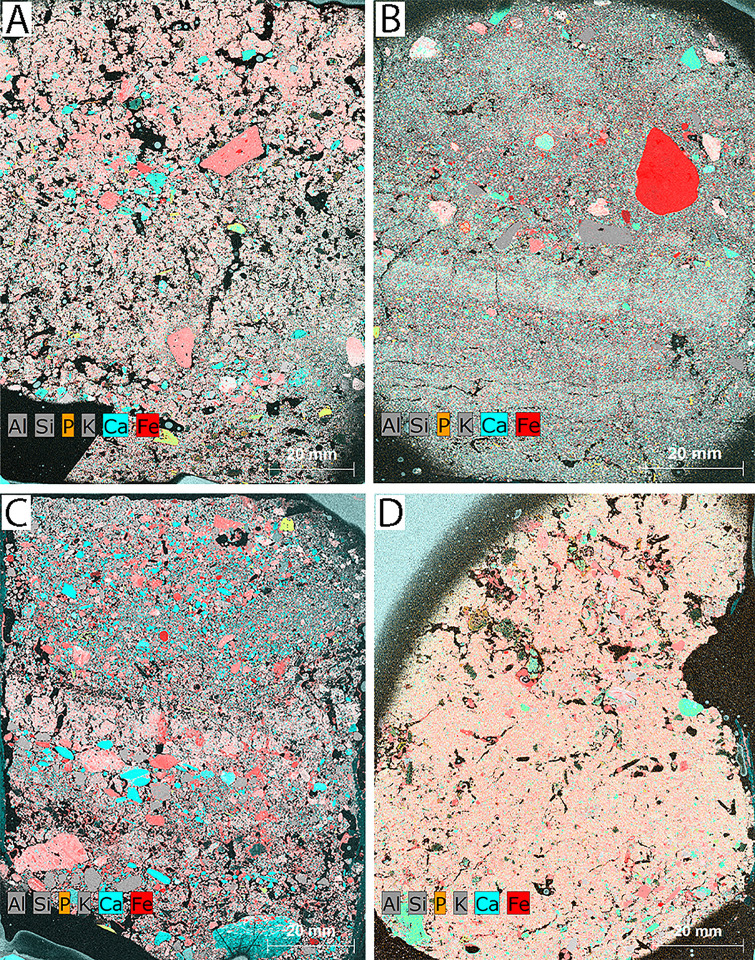
Micro-XRF elemental maps of thin sections from sewer contexts: (A) Sample 4, (B) Sample 5, (C) Sample 7, and (D) Sample 8 showing elemental distributions of Al, Si, K, P, Ca, and Fe. Al, Si and K are enriched in geogenic components and terra cotta fragments and here are represented in grey. Ca is present in both limestone and lime, which appear as aqua. P is represented in orange, but in these samples is nearly always present in association with Ca within amorphous Ca-phosphates, authigenic hydroxylapatite, and bone yielding a yellow color in the map. Fe (likely iron oxide) is represented in red; when co-occurring with Si, Ka or Al these areas appear pink (Credit: Susan M. Mentzer).

In Sample 4 (sewer), the distribution of Al and Si is homogeneous and represents the geogenic sediment matrix. Ca and P occur in more discrete, discontinuous bands, whereas Fe form localized halos around particles, suggesting oxidizing post-depositional conditions (Fig 11A). Compared to Sample 2, the lamination is more diffuse, indicating gradual and intermittent sedimentation.

Sample 5 (sewer) displays moderate Ca–P co-association within finer matrix zones and near ceramic inclusions (Fig 11B). Fe forms dispersed oxidation halos, and although laminae are faint, they remain recognizable. The elemental configuration suggests phased inputs combined with localized post-depositional alteration. Al and Si remain evenly distributed, confirming the dominance of natural sediment influx.

Sample 7 (sewer) shows localized Ca and P enrichments, but these do not form distinct or rhythmic elemental bands (Fig 11C). Ca, P, and Fe occur in small, discontinuous patches along internal boundaries, indicating subtle and irregular geochemical variability within the sample. While micromorphology reveals a clear coarse–fine–charcoal alternation, the micro-XRF elemental signals do not mirror this stratigraphic rhythm.

Finally, Sample 8 (sewer) exhibits Ca and P in less structured zones with fewer well-defined layers (Fig 11D). P is localized but weakly expressed, and reacted lime is present but scattered. Ceramic fragments are abundant. Compared to Sample 7, lamination is less distinct and Fe concentrations more unevenly distributed, suggesting wetter, more reductive conditions and enhanced organic decay.

### PED-XRF

PED-XRF analysis provides complementary geochemical data to the micromorphological and micro-XRF results by offering bulk compositional profiles from latrine and sewer fill (S4 Table). This method allows for the statistical evaluation of depositional variability, anthropogenic input, and chemical differentiation on a broader scale [43].

**Fig 12:**
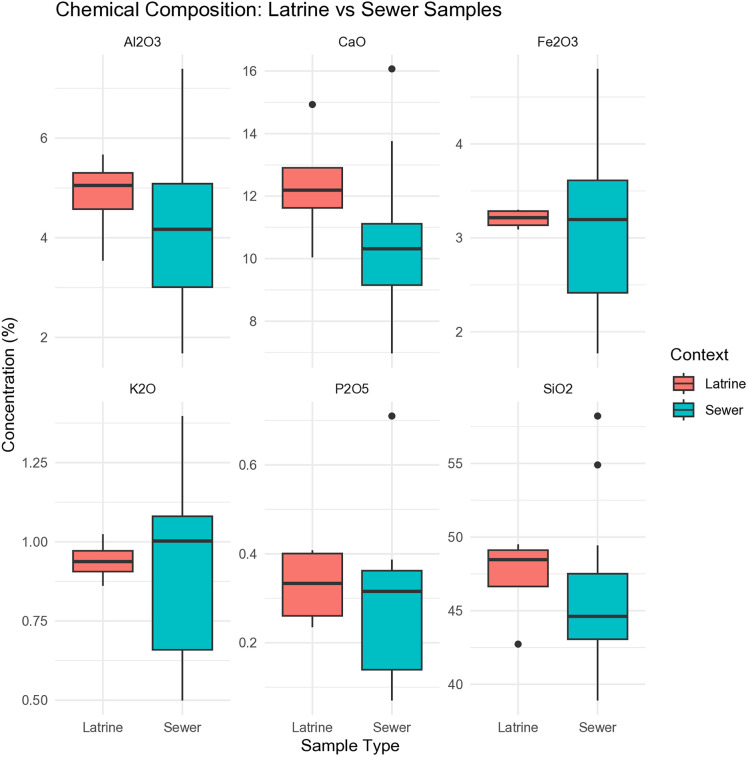
Boxplots illustrating the distribution of major oxides (weight %) in latrine and sewer samples (Credit: Melis Uzdurum).

Boxplots show that latrine samples are enriched in P₂O₅ and CaO, supporting interpretations of lime addition and fecal deposition (Fig 12). In addition, SiO₂ and Al₂O₃ values are clearly higher in the latrine samples than in the sewer fills, indicating a greater contribution of fine, silicate-rich material to the latrine deposits. In contrast, the sewer samples display lower SiO₂ and Al₂O₃ values and greater variability in Fe₂O₃, which likely reflects fluctuating hydrological conditions and post-depositional oxidation processes within the drainage system.

**Fig 13:**
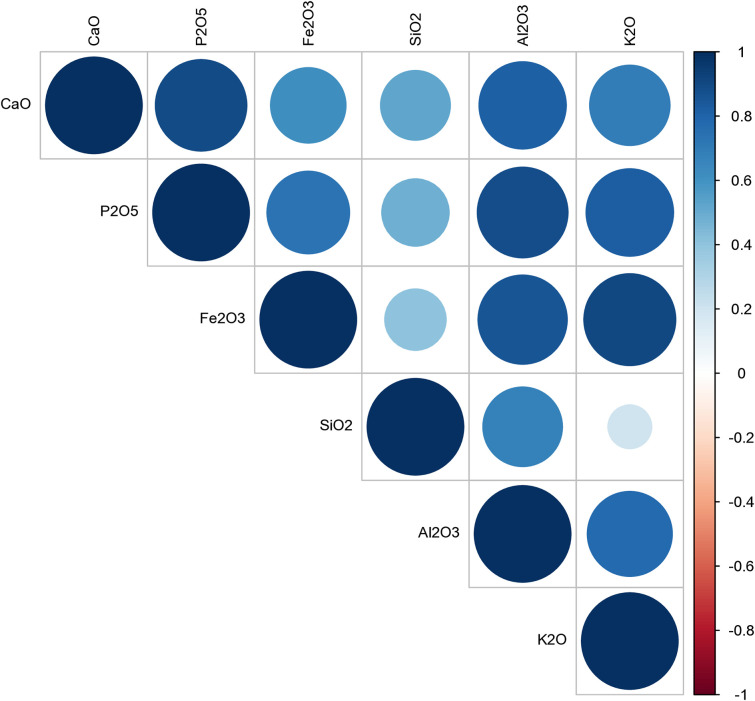
Correlation matrix of major oxides showing relationships between CaO, P₂O₅, SiO₂, Al₂O₃, Fe₂O₃, and K₂O across all samples (Credit: Melis Uzdurum).

Correlational analysis identifies strong positive relationships between CaO and P₂O₅, indicative of lime-phosphate interaction, and between SiO₂ and Al₂O₃, consistent with shared sedimentary origins (Fig 13). Negative correlations between CaO and silicate oxides may reflect both anthropogenic lime-rich inputs and the presence of geogenic limestone fragments identified.

**Fig 14:**
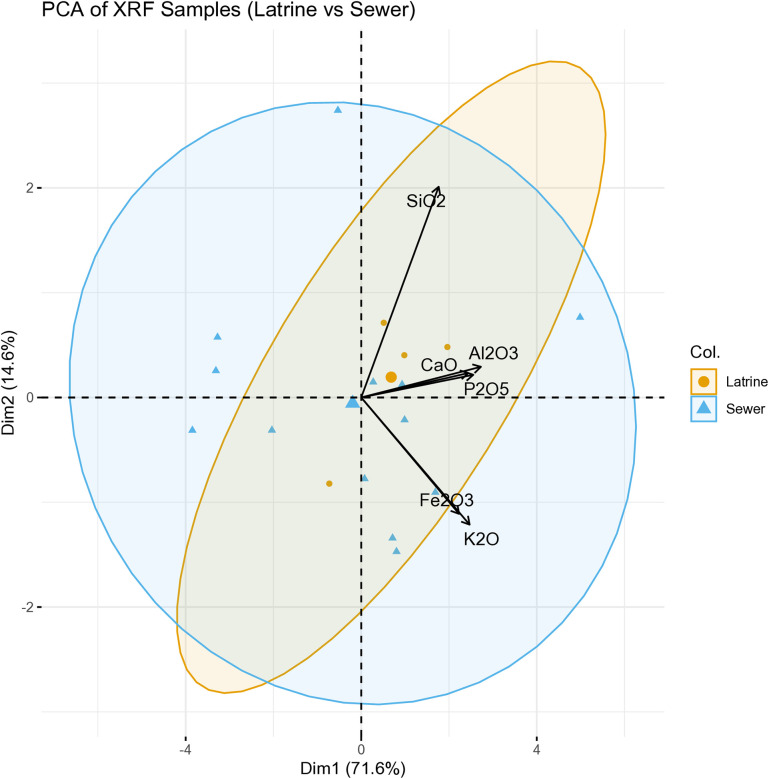
PCA biplot of XRF data from latrine and sewer samples, based on six major oxides (CaO, P₂O₅, SiO₂, Al₂O₃, K₂O, Fe₂O₃) (Credit: Melis Uzdurum).

PCA was performed on six selected elements accounting for 86.2% of the total variance (PC1: 71.6%, PC2: 14.6%). The biplot reveals a structured chemical separation between contexts (Fig 14). Latrine samples cluster tightly along the positive axis of PC1, strongly associated with CaO and P₂O₅ loadings. This pattern reflects consistent lime addition and organic input in line with sanitation-related use. In contrast, sewer samples display broader dispersion across both PC1 and PC2, reflecting greater compositional heterogeneity. This spread is driven by more variable SiO₂, Al₂O₃, and Fe₂O₃ values, likely reflecting fluctuating waterborne inputs and sedimentary processes. The clear axis-aligned positioning of latrine samples versus the scattered distribution of sewer samples underscores different formation dynamics: intentional and repeated inputs in the latrine versus episodic, mixed-source deposition in the sewer.

**Fig 15:**
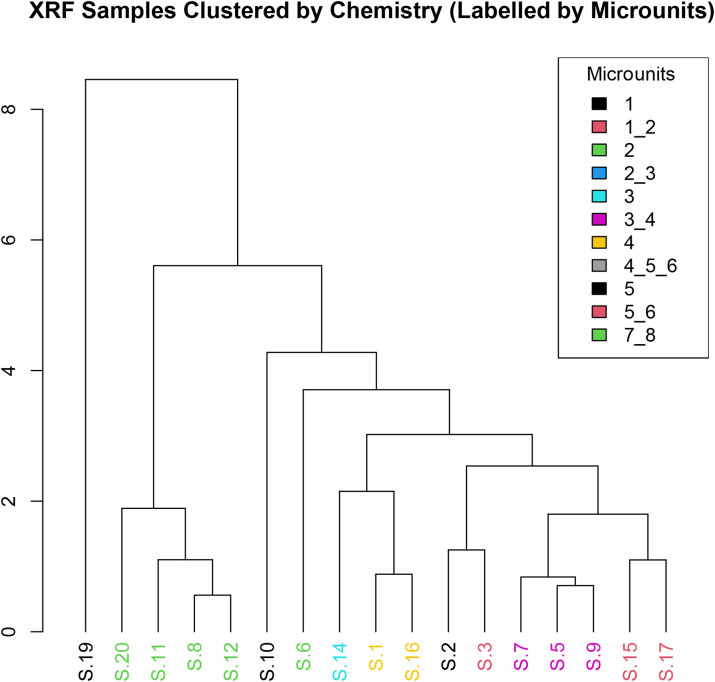
HCA of elemental composition (based on mean values), illustrating chemical affinities among microunits across contexts (Credit: Melis Uzdurum).

HCA reveals that certain microunits exhibit strong compositional similarities regardless of their broader context (Fig 15). For example, S.2_microunit 2 (latrine) clusters with S.19_microunit 1 (sewer), both characterized by elevated CaO and P₂O₅ concentrations, suggesting similar inputs likely related to lime and phosphate-rich deposits. In contrast, S. 4_microunits 4 and S.5_microunit 1 are grouped based on low CaO and high SiO₂–Al₂O₃ levels, pointing to a stronger geogenic sediment signature. Microunits from the same thin section—such as S.7_microunits 2–3 and S.7_microunits 7–8—fall into different clusters, highlighting intra-sample heterogeneity and episodic sediment deposition within the sewer context.

**Fig 16:**
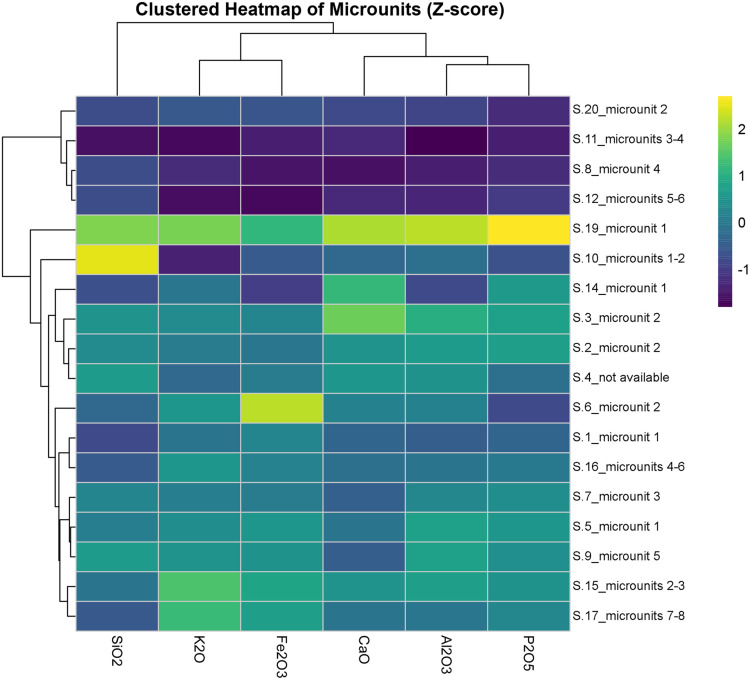
Clustered heatmap of microunits based on mean elemental concentrations (SiO₂, Al₂O₃, K₂O, CaO, Fe₂O₃, P₂O₅), normalized as Z-scores (Credit: Melis Uzdurum).

The clustered heatmap confirms that latrine microunits are compositionally more consistent and enriched in CaO and P₂O₅. S.1_microunit 1, S.2_microunit 2, and S.10_microunit 2, for instance, form a distinct cluster, likely reflecting inputs rich in lime and organic waste (Fig 16). Conversely, sewer microunits such as S.4_microunit 3 and S.7_microunits 4–6 cluster separately due to higher SiO₂ and Al₂O₃ values, consistent with sedimentation driven by flowing water. The grouping of S.5_microunit 1 with S.7_microunit 3 also points to recurring depositional patterns in mid-sewer contexts. These results highlight that microunit-level compositional differences are not only context-specific but also stratigraphically structured within individual features.

### Interpretation, integration and discussion on the proxy data

#### Sanitation practices and human activity in the latrine and sewer contexts.

Multiproxy data were integrated to address specific research questions concerning the intensity and nature of anthropogenic activity. Archaeobotanical results highlight the presence of both cultivated and wild plant taxa within the sewer and latrine deposits. Charred wheat (*Triticum sp.*), barley (*Hordeum sp.*), and grape (*Vitis vinifera*) found in both contexts are consistent with phases of concentrated human activity, particularly food processing and waste disposal [21,24]. However, the parallel dominance of non-cultivated wild taxa composed of both ruderal and segetal groups should not only be interpreted as natural intruders but also due to human activities, either as cultivation and disturbed soils and waste locations. Their consistent presence, especially in later stratigraphic levels, points to a transformation of the urban space into a more rural or unmanaged character during the final use and post-abandonment phases. Rich variety (23 different) and number of weeds indicate regeneration of wild flora and increasing rural character of the settlement considering many of them as crop weeds, but majority as ruderals (for the interpretation of weeds as part of the past environment, medicinal use and agricultural economy [58]). This pattern likely reflects a gradual withdrawal of anthropogenic input, replaced over time by environmental re-colonization. Therefore, we consider the presence of the wild taxa with human activity but also entered in the archaeological context after the abandonment of the site.

A chronological distinction between the two features supports gradual transformation of the Domus within the urban setting. The latrine appears to have gone out of use by the 6th century, whereas the sewer remained active until at least the second half of the 7th century. During this transformation, the continuous presence of wild seeds within the feature deposits indicates that the area was developing into a more rural environment through either cultural or natural formation processes.

Micromorphological analysis show that the latrine represents a closed, short-term depositional event marked by deliberate sanitation activity, lime application, and episodic waste input, whereas the sewers reflect sustained use into the 7th century CE. Layering patterns, oxidation features, and phosphate–lime associations indicate a dynamic, intermittently maintained urban drainage system. These contrasting micromorphological signatures illustrate asynchronous trajectories of domestic and public spaces at Pompeiopolis: private facilities were abandoned or repurposed earlier, while public drainage remained active longer—findings also confirmed by the radiocarbon results.

Microscopically identifiable human excrement is rarely preserved in archaeological contexts [59,60]. While coprolites have been recorded in caves, middens, and dedicated latrines [61], their intact preservation generally occurs only in arid environments. In most latrine contexts, excrement breaks down rapidly and survives only in partial or mineralized forms [38]. For this reason, indirect indicators—such as amorphous phosphatic phases under oxygenated conditions or vivianite in anoxic ones—are more reliable [38,62]. Molecular proxies such as coprostanol have also been successfully used to detect human fecal input in sediments [63]. Phosphorus, being highly concentrated in fecal waste, serves as a key elemental proxy [64], and phosphate enrichment along sediment interfaces or within voids often signals leakage from cesspits or drains. Secondary Ca–P precipitates—particularly phosphatic nodules [65] and crusts [66]—can serve as robust macroscopic and microscopic proxies for dung accumulation and penning activity, highlighting the broader interpretive value of phosphate-based indicators in feces-rich deposits.

In the latrine context at Pompeiopolis, multiple lines of evidence support this interpretation. Micro-XRF mapping provides important spatial information, but it also shows a pattern that requires careful contextualization: although fecal material was likely present, phosphorus intensities remain generally low and are detectable mainly in localized Ca–P coatings. This behavior most closely resembles dung-rich contexts where deposits have been strongly modified by taphonomic and diagenetic processes—such as the Middle Bronze Age stabling layers [67] and dung-rich deposits from both waterlogged and well-drained Bronze Age sites recently documented through micro-XRF analysis [68]. In these specific cases, phosphorus appears weak in micro-XRF maps except within discrete phosphatic crusts or hypocoatings. Importantly, however, this pattern does not characterize the majority of archaeological dung deposits, which typically retain high levels of phosphorus and often produce strong micro-XRF signals due to limited decomposition. In the Pompeiopolis latrine fill, Ca and P co-occur only in limited spots—likely representing mineralized residues of organic waste interacting with lime—while the broader matrix shows no strong P signature, consistent with a deliberately managed waste context designed to promote thorough organic breakdown and ammonia neutralization.

Additional support comes from the archaeobotanical record. Although whole excrement is absent, the archaeobotanical record offers another layer of support. A single *Rubus fruticosus* (blackberry) seed, a key indicator of cesspit deposits [69], was found in the latrine fill (US 1806), could support the identification of the latrine function. This, alongside fig and grape seeds, further supports the identification of a cesspit-like function for the latrine.

The sedimentological properties of the latrine also contribute to this interpretation. The fill is composed primarily of sandy material, lacking concentrated organic mass. This is consistent with the expected transformation of excrement into phosphate minerals under archaeological conditions [60,62]. While latrine layers exhibit high phosphate values, organic decomposition in the sewer is present to a lesser degree, with lower phosphorus levels (0.100–0.375%). However, the micro-XRF maps show only low and localized P signals, a pattern that corresponds to dung-rich contexts where decomposition is already advanced, rather than to the majority of archaeological dung deposits, which typically show strong and widespread phosphorus enrichment. Charcoal-rich layers, such as S.7_microunit 2, signal episodic waste-related inputs, possibly reflecting disposal events or runoff from adjacent areas.

Micromorphological and micro-FTIR analyses (see Section 3.2 for details) add diagnostic resolution: lime fragments in the latrine context show partial reaction with amorphous Ca–P phases, and these P-rich precipitates are encased in calcite—a likely result of lime exposure. This mineralogical association highlights the deliberate addition of lime to fecal waste, a historically widespread method for reducing odor and enhancing hygiene [70,71]. Such practices reflect intentional latrine design aimed at controlling decomposition and minimizing olfactory discomfort.

Comparable waste management strategies are documented elsewhere in the Roman world (see [72,73] for earlier periods). At the ‘Imperial Baths’ in Sagalassos, archaeobotanical and biomarker studies revealed human feces in the sewer channel and herbivore dung on the latrine floor. These were interspersed with lime layers, indicating systematic sanitation measures [74]. The parallel evidence from Pompeiopolis—lime-treated waste, phosphate accumulation, and contextual botanical signals—illustrates how sanitation infrastructure was actively managed during the final urban phases before abandonment.

#### Environmental succession and the material traces of urban decline.

Beyond the evidence for sanitation-related practices, the integration of macrobotanical and geochemical data offers valuable insights into the broader transformation of the built environment at Pompeiopolis. A clear shift can be observed between cultivated and wild taxa across the stratigraphic units. While cultivated taxa—such as wheat, barley, and grape—appear in limited quantities, primarily in the latrine, wild taxa are significantly more abundant and widespread, particularly in the sewer deposits. This disproportion suggests that human activity had diminished considerably by the time upper sewer fills accumulated. The increase in taxonomic variety (23 wild species), coupled with their vertical distribution, underscores a transition from managed space to environmentally driven deposition.

The rich variety and number of wild taxa are significant for two reasons. First, they indicate the ruralization of the settlement’s surroundings. Sagittal species such as *Polycnemum* sp., *Trifolium* sp., and *Galium* sp. persist in fields alongside cultivated crops, while ruderal species like *Chenopodium* sp., *Polygonum* sp., and *Heliotropium* sp. rapidly reproduce in disturbed areas, midden zones, and ruined spaces [51–53]. In addition, species such as *Fumaria* sp. (antispasmodic and anticeptic), *Heliotropium* sp. (pyrrolidizine alkaloid against poisonous bites) and *Lithospermum* sp. (contraception) are known to have been among the main plants used in medical treatments and other uses (*Fumaria* sp. as a dye) in the past [58]. From this perspective, we can speak of a strong interaction between humans and the natural environment at the settlement, and thus of a process of ruralization.

Secondly, the diversity among wild species provides information about the past natural environment and potential habitats during the settlement became rural and was subsequently abandoned, offering valuable data for reconstructing the past landscapes (i.e., *Polycnemum* sp. grows in sand and stony fields, *Chenopodium* sp. in fallow or cultivated fields, *Lithospermum* sp. in limestone embarkments, clayey soils, arable field boundaries and rocky places) [58]. These indicators vice versa support the geological formations around the site. Overall, the evidence suggests that ruralization and the subsequent ecological regeneration following abandonment occurred gradually during the 6th and 7th centuries.

The sewer system, in particular, reflects this shift from an engineered drainage network to a passive depositional basin. The upper microunits of the sewer (e.g., S.7,_microunit 1 and S 8,_microunit 1) provide compelling evidence for this transformation. XRF data show significantly elevated concentrations of CaO (16.07%) and SiO₂ (54.90%), which likely correspond to the influx of construction debris such as lime-treated wall material and silicate-rich sediments (e.g., ceramics, sand). These materials were probably introduced as sedimentation control diminished following the cessation of regular maintenance.

Micromorphological observations support this interpretation. Coarser grain sizes and less structured laminae in the upper layers indicate uncontrolled infill processes, contrasting sharply with the well-layered, high-energy deposits of the sewer’s active phase. In these later contexts, secondary accumulation of carbonates and silicates suggests gradual sedimentation under fluctuating hydrological conditions, possibly reflecting intermittent runoff or surface erosion. This pattern is further emphasized by the weakening of lamination in Sample 8, alongside scattered Fe distribution—markers of wetter, more reductive post-depositional environments and organic degradation.

Additionally, Fe₂O₃ variation across sewer samples highlights the chemical consequences of infrastructure abandonment. The presence of secondary iron oxide accumulations suggests periodic oxidation–reduction cycles, possibly resulting from fluctuating groundwater levels or variable exposure to air. These elemental signals reinforce the interpretation of the sewer as an increasingly open and unmanaged depositional feature.

From a compositional standpoint, cluster analysis also reveals context-transcending patterns. For instance, the similarity between latrine S.2,_microunit 2 and sewer S.19,_microunit 1—both showing elevated CaO and P₂O₅—suggests that lime-rich deposits may have originated from comparable waste or construction materials, regardless of architectural context. This highlights the widespread presence of similar depositional inputs across both private and public domains during the post-urban phase.

As a whole, these findings emphasize how environmental indicators—wild plant assemblages, mineral deposits, sediment textures, and chemical signatures—can serve as proxies for social and spatial abandonment. In the absence of active management, nature reasserts itself within the archaeological record, leaving stratified traces of gradual urban decline and ecological succession. The city’s decline was not abrupt, but played out through layered transformations—first social, then spatial, and finally environmental.

## Conclusion

This study applied a multiproxy, macro-and-micro-scale approach to assess the dynamics of urban transformation in Late Antique Pompeiopolis, focusing on how sanitation infrastructure and waste-related features reflected shifts in human behavior, environmental processes, and maintenance patterns. By integrating micromorphology, geochemistry, and archaeobotany, we reconstructed the divergent trajectories of a private latrine and a public sewer during the 6th and 7th centuries.

The results reveal that the latrine was decommissioned and rapidly infilled by the 6th century, whereas the sewer continued to function into the second half of the 7th century. High carbonate and phosphate concentrations in the latrine deposits indicate deliberate sanitation practices and active waste management. In contrast, coarse grain sizes and high-energy depositional patterns in the lower sewer levels reflect its prolonged active use. The upper layers of the sewer, however, show increased wild macrobotanical taxa and finer sedimentation, suggesting ecological succession and the gradual decline of anthropogenic input.

These findings highlight how different infrastructural components responded to urban change at different tempos. Micromorphological observations further supported these trajectories, revealing lime-treated waste in the latrine and rhythmic coarse-to-fine layering in the sewer, reflecting episodic runoff and fluctuating maintenance. The sewer transformed from a functional system into an anthropogenically passive depositional feature, shaped increasingly by natural processes after human activity ceased. These localized processes reflect broader urban restructuring at Pompeiopolis, including the subdivision of elite residences, the spatial contraction of the settlement, the disintegration of elite space, the decline of public services, and the ruralization of the urban fabric.

By documenting the interplay between cultural and natural formation processes, this study contributes to a broader understanding of Late Antique–Early Byzantine urban transformation in Anatolia. The integration of micromorphological, chemical, microdebris, and macrobotanical data provides a robust interpretive framework for identifying shifts in human activity and environmental response. In doing so, the study not only enhances our knowledge of Pompeiopolis but also offers a comparative model for analyzing similar post-urban contexts across comparable urban situations in Anatolia.

## Supporting information

S1 Table C14 Results. The results are registered as Tübitak MAM Report No. 29109288-125.05-3896/26897.
(XLSX)

S2 Table Identified plant species (count/liter). (XLSX)

S3 Table Microdebris (Heavy Residue) artifacts (count/liter). (XLSX)

S4 Table PED-XRF results. (XLSX)
